# MiRNA-Related Genetic Variations Associated with Radiotherapy-Induced Toxicities in Patients with Locally Advanced Non–Small Cell Lung Cancer

**DOI:** 10.1371/journal.pone.0150467

**Published:** 2016-03-18

**Authors:** Rong Li, Xia Pu, Joe Y. Chang, Yuanqing Ye, Ritsuko Komaki, John D. Minna, Jack A. Roth, Baohui Han, Xifeng Wu

**Affiliations:** 1 Department of Epidemiology, The University of Texas MD Anderson Cancer Center, Houston, Texas, United States of America; 2 Department of Pulmonary Medicine, Shanghai Chest Hospital, Shanghai Jiao Tong University, West Huaihai Road 241, Shanghai, China; 3 Department of Radiation Oncology, The University of Texas MD Anderson Cancer Center, Houston, Texas, United States of America; 4 Hamon Center for Therapeutic Oncology Research, University of Texas Southwestern Medical Center at Dallas, Dallas, TX, United States of America; 5 Department of Thoracic and Cardiovascular Surgery, The University of Texas MD Anderson Cancer Center, Houston, Texas, United States of America; University of Barcelona, SPAIN

## Abstract

Severe radiation-induced toxicities limit treatment efficacy and compromise outcomes of lung cancer. We aimed to identify microRNA-related genetic variations as biomarkers for the prediction of radiotherapy-induced acute toxicities. We genotyped 233 SNPs (161 in microRNA binding site and 72 in processing gene) and analyzed their associations with pneumonitis and esophagitis in 167 stage III NSCLC patients received definitive radiation therapy. Sixteen and 11 SNPs were associated with esophagitis and pneumonitis, respectively. After multiple comparison correction, *RPS6KB2*:rs10274, *SMO*:rs1061280, *SMO*:rs1061285 remained significantly associated with esophagitis, while processing gene *DGCR8*:rs720014, *DGCR8*:rs3757, *DGCR8*:rs1633445 remained significantly associated with pneumonitis. Patients with the AA genotype of *RPS6KB2*:rs10274 had an 81% reduced risk of developing esophagitis (OR: 0.19, 95% CI: 0.07–0.51, p = 0.001, q = 0.06). Patients with the AG+GG genotype of *SMO*:rs1061280 had an 81% reduced risk of developing esophagitis (OR: 0.19, 95% CI: 0.07–0.53, p = 0.001, q = 0.06). Patients with the GG+GA genotype of *DGCR8*:rs720014 had a 3.54-fold increased risk of pneumonitis (OR: 3.54, 95% CI: 1.65–7.61, p <0.05, q <0.1). Significantly cumulative effects of the top SNPs were observed for both toxicities (P-trend <0.001). Using bioinformatics tools, we found that the genotype of rs10274 was associated with altered expression of the *RPS6KB2* gene. Gene-based analysis showed *DGCR8* (p = 0.010) and *GEMIN4* (p = 0.039) were the top genes associated with the risk of developing pneumonitis. Our results provide strong evidence that microRNA-related genetic variations contribute to the development of radiotherapy-induced acute esophagitis and pneumonitis and could thus serve as biomarkers to help accurately predict radiotherapy-induced toxicity in NSCLC patients.

## Introduction

Non-small cell lung cancer (NSCLC) accounts for 85% of all lung cancer cases [1]; approximately one fifth (20%) of NSCLC patients have locally advanced (stage III) disease at the time of diagnosis [2] and have a poor 5-year survival rate (lower than 14%) [3]. Radiotherapy combined with chemotherapy is the mainstay for locally advanced NSCLC [4]. It can enhance control of local disease and to improve 5 year survival rate by 4.5% [5–6]. However, radiation-induced toxicity to normal tissue often limits the efficacy of definitive radiation therapy. Severe acute esophagitis and symptomatic pneumonitis occur in approximately 15–25% and 5–50% of NSCLC patients, respectively [7–8]. These toxic effects are dose-limiting and can compromise treatment outcomes.

Currently, clinical and dosimetric factors are used to predict the possibility of radiation-induced toxicities and to guide dose design [9–10]. However, these clinical factors lack sufficient accuracy in the prediction of these toxicities, with limited negative predictive values (60% – 80%) and high false-negative rates (25% – 50%) [11]. Therefore, there is a strong need to identify novel biomarkers to assist in the accurate prediction of these toxicities and to guide radiation dose design before treatment. Genetic variations appear to be promising biomarkers, as they have been associated with radiotherapy toxicities in many studies [12–14]; however most of these studies focused on only a limited number of genes.

MicroRNA (miRNA) is a class of small non-coding RNAs that regulates gene expression by binding to the target mRNA. MiRNAs play an important role in cancer development and therapeutic responses [15–16]. Evidence has linked miRNAs with radiation-induced side effects such as hematopoietic injury [17]. Previous study has also reported that genetic variation within miRNA processing genes or miRNA-mRNA binding sites could influence miRNA maturation or regulation [18] and were associated with clinical outcomes in NSCLC patients [19].

In this study, we analyzed 161 SNPs within predicted microRNA binding sites genes in major cancer-related genes and 72 SNPs in microRNA processing genes. We evaluated the relationship between these variants and radiation-induced pneumonitis and esophagitis in patients with locally advanced NSCLC treated with definitive radiation therapy. Using Silicon bioinformation queries (Silicon Genetics), we functionally characterized the potential mechanism of identified loci. To our knowledge, ours is the first effort to comprehensively evaluate the effect of miRNA-related genetic variations on the risk of developing radiation-induced toxicities.

## Material and Methods

### Ethics statement

This study was approved by the institutional review board (IRB) of MD Anderson Cancer Center (Houston, TX), and written informed consent was obtained from all participants according to procedures approved by IRB.

### Study population and data collection

All patients had histologically confirmed, newly diagnosed NSCLC and were recruited from MD Anderson Cancer Center between September 1995 and February 2008. Patient eligibility criteria for the study were locally advanced stage III NSCLC, Caucasian race, treated with primary radiation therapy (with or without chemotherapy). In addition, for cases treated with concurrent chemoradiotherapy, all were subjected to platinum-based chemotherapy. Clinical stage was defined according to American Joint Committee on Cancer (AJCC) staging system (version 6).

A questionnaire was used to collect epidemiologic data during an in-person interview. Blood sample of patients were drawn from each patient. Clinical variables and follow-up information were abstracted from medical records. Radiation-induced toxicities were defined following the criteria defined previously (19). Briefly, we used documentation of new-onset pain upon swallowing that occurred during treatment to define esophagitis and we used roentgenographic or CT scan abnormalities that were often associated with nonproductive cough and/or fever to detect pneumonitis. Radiation-induced toxicities graded according to National Cancer Institute Common Terminology Criteria for Adverse Events (version 3.0) guidelines [19]. We defined an event as the occurrence of a severe (grade ≥2) pneumonitis or esophagitis.

### SNP selection and genotyping

The details of the SNP selection were described previously [20,21]. Briefly, we previously developed a panel that included major genes in cancer-related pathways [21], among which there were seven microRNA processing genes. Tagging SNPs (±10 kb flanking each gene) and potential functional SNPs for each gene were included. MiRNA binding site SNPs were identified using the PolymiRTS v3.0 [22] for the genes included on the chip. Genomic DNA was extracted from the study patients’ peripheral blood using the Human Whole Blood Genomic DNA Extraction Kit (Qiagen, Valencia, CA). Genotyping was performed using Custom Illumina iSelect Infinium II Beadchips following the standard protocol (Illumina, San Diego, CA). Only SNPs with a sample call rate greater than 95% and samples with an SNP call rate greater than 95% were included in the final analysis.

### Statistical analysis

Chi-square test, Fisher’s exact test and student’s t-test were used to analyze the distribution of demographic and clinical variables between patients with or without toxic reactions. Multivariate logistic regression was used to assess the main effect of single SNP on the risk of developing pneumonitis or esophagitis, adjusted for patient age, sex, performance status, smoking status, clinical stage of disease, radiation therapy type, chemotherapy, radiation dosimtric variables, and lung function. A complete analysis of the effect of all 233 individual SNPs on risk of esophagitis and pneumonitis are shown in S1 Table and S2 Table, respectively. Multiple hypothesis testing adjustment was performed using the "q-value" package in R software [23] based on a false discovery rate of 10% that has been used in prior studies of clinical outcomes [24–26]. Cumulative effects were analyzed by calculating unfavorable genotypes (UFGs), which were defined as counting the number genotypes from the identified SNPs (p<0.01) associated with an increased risk of developing radiation-induced toxicity. All the analyses above were performed using STATA software (version 10, STATA Corp., College Station, TX). Gene-based analysis was performed using Versatile Gene-Based Association Study software (VEGAS) [27]. Expression Quantitative Trait Loci (eQTL) analysis was conducted using the Genevar [28] (GENe Expression VARiation) database(http://www.sanger.ac.uk/resources/software/genevar/). The results were based on data for the Multiple Tissue Human Expression Resource (MuTHER) Study [29]. The potential miRNA binding site SNPs were identified using PolymirTS v1.0 (Table A and Table B in S1 File). Spearman’s correlations were obtained for SNPs and the corresponding mapped genes to perform cis-eQTL. A two-side value of p<0.05 was considered statistically significant.

## Results

### Host characteristics

A total of 167 patients were included in this study; 71% of patients had grade 2 or higher esophagitis, 38% of patients had grade 2 or higher pneumonitis, and 28% of patients had both esophagitis and pneumonitis (Table 1). There was no significant difference between patients with or without severe toxicities for both esophagitis and pneumonitis in terms of age, sex, smoking pack years, Carbon Monoxide Diffusing Capacity (DLCO), expiratory volume in 1 second (FEV1), planning target volume (PTV), clinical stage of disease, performance status or chemotherapy. The majority of the patients (>95%) were treated with concurrent, platinum-based chemoradiotherapy. Both radiation dosimetrics and radiotherapy type exhibited significant difference between patients with or without esophagitis. Mean esophageal dose was significantly higher for patients with esophagitis (36.79±10.69Gy) than those without esophagitis (30.62±9.32Gy) (p = 0.04). Mean lung radiation dose for patients with pneumonitis was significantly higher (23.03±11.98Gy) than those without pneumonitis (18.86±5.22Gy) (p = 0.01).

**Table 1:** Characteristics of Patients with Stage III NSCLC by Esophagitis and pneumonitis Status.

Characteristics	Esophagitis Status			Pneumonitis Status		
	Event	No event	*p*	Event	No event	*p*
**Age, mean(SD)**	60.65 (10.04)	62.83 (9.94)	0.203	62.55 (9.46)	60.63 (9.97)	0.234
**Smoking Pack year, mean(SD)**	47.87 (29.63)	42.41 (24.44)	0.259	47.32 (32.04)	45.05 (24.37)	0.616
**DLCO(mL), mean(SD)**	70.32 (16.90)	70.29 (22.70)	0.997	67.67 (15.25)	72.54 (21.24)	0.441
**FEV1(mL), mean(SD)**	73.96 (17.52)	71.59 (19.74)	0.682	72.13 (15.34)	73.62 (20.41)	0.803
**PTV(mL), mean(SD)**	908.50 (504.64)	728.21 (452.80)	0.106	844.00 (561.11)	846.57 (473.85)	0.982
**Mean Esophageal Dose (Gy) mean(SD)**	36.79 (10.69)	30.62 (9.32)	0.004*	35.52 (11.76)	34.25 (10.38)	0.569
**Mean Lung Dose (Gy) mean(SD)**	21.31 (9.3)	18.05 (4.4)	0.048	23.03 (11.98)	18.86 (5.22)	0.015*
**Sex**						
** Male, n (%)**	64 (54)	27 (56)	0.772	31 (52)	53 (55)	0.717
** Female, n (%)**	55 (46)	21 (44)		29 (48)	44 (45)	
**Clinical Stage of Disease**						
** IIIA, n (%)**	64 (54)	28 (58)	0.593	36 (60)	52 (54)	0.432
** IIIB, n (%)**	55 (46)	20 (42)		24 (40)	45 (46)	
**Performance status**						
** 0, n (%)**	41 (34)	10 (21)	0.161^	18 (30)	31 (32)	0.417^
** 1, n (%)**	52 (44)	27 (56)		29 (48)	43 (44)	
** 2~4, n (%)**	8 (7)	6 (13)		3 (5)	11 (11)	
** Missing, n (%)**	18 (15)	5 (10)		10 (17)	12 (12)	
**Chemotherapy**						
** concurrent chemoradiotherapy, n (%)**	117 (98)	44 (92)	0.057^	58 (97)	93 (96)	1^
** Others, n (%)**	2 (2)	4 (8)		2 (3)	4 (4)	
**Radiotherapy type**						
** 2D, n (%)**	31 (26)	12 (25)	0.021^*	14 (23)	28 (29)	0.578^
** 3D, n (%)**	56 (47)	14 (29)		29 (48)	36 (37)	
** IMRT, n (%)**	29 (24)	16 (33)		15 (25)	27 (28)	
** Proton, n (%)**	3 (3)	6 (13)		2 (3)	6 (6)	

DLCO: Carbon Monoxide Diffusing Capacity

FEV1: expiratory volume in 1 second

PTV: planning target volume

IMRT: Intensity-modulated radiation therapy

^ Fisher's exact test

*p<0.05.

### Associations between individual SNPs and radiation-induced acute esophagitis

In all, 233 SNPs (161 SNPs from predicted microRNA binding sites and 72 SNPs from microRNA processing sites) were included in the analysis. We found 16 SNPs significantly associated with a risk of developing esophagitis at p<0.05 (Table 2), including 13 SNPs located in microRNA binding sites and 3 SNPs in microRNA processing genes. The predictive miRNAs that may potentially target these binding sites are listed in Table A in S1 file. After multiple comparison corrections, three SNPs (*RPS6KB2*:rs10274, *SMO*:rs1061280, *SMO*:rs1061285) remained significantly associated with esophagitis (q<0.1) (Table 2). Patients with the AA genotype of *RPS6KB2*:rs10274 has an 81% reduced risk of developing esophagitis (OR: 0.19, 95% CI: 0.07–0.51, p = 0.001, q = 0.06). Patients with AG+GG genotype of *SMO*:rs1061280 (in high LD with *SMO*:rs1061285, *r*^2^>0.8) had an 81% reduced risk of developing esophagitis (OR: 0.19, 95% CI: 0.07–0.53, p = 0.001, q = 0.06).

**Table 2:** Association of Esophagitis with the top 16 MicroRNA SNPs.

SNP	Gene	Site	Chr	Allelic change	Model*	OR (95% CI)^#^	*p*
rs10274	RPS6KB2	binding	11	G>A	rec	0.19 (0.07–0.51)	0.001^
rs1061280	SMO	binding	7	A>G	dom	0.19 (0.07–0.53)	0.001^
rs1061285	SMO	binding	7	C>A	dom	0.19 (0.07–0.53)	0.001^
rs3124591	NOTCH1	binding	9	A>G	add	0.39(0.20–0.75)	0.005
rs1133043	GPR30	binding	7	C>G	rec	0.16(0.04–0.64)	0.01
rs16950113	SMAD7	binding	18	A>G	dom	0.23(0.07–0.75)	0.015
rs713065	FZD4	binding	11	G>A	add	2.36(1.14–4.89)	0.02
rs2740351	GEMIN4	processing	17	A>G	add	2.15(1.12–4.12)	0.022
rs7813	GEMIN4	processing	17	A>G	add	2.15(1.12–4.12)	0.022
rs2075993	E2F2	binding	1	A>G	rec	0.32(0.11–0.90)	0.03
rs7588	PLK1	binding	16	G>A	dom	0.40(0.17–0.92)	0.031
rs573010	RNASEN	processing	5	C>A	rec	0.14(0.02–0.84)	0.031
rs1052133	OGG1	binding	3	C>G	add	2.34(1.07–5.10)	0.033
rs4690150	FGF5	binding	4	C>G	dom	2.53(1.06–6.01)	0.036
rs2248718	ATP6V1C1	binding	8	G>A	add	0.49(0.25–0.98)	0.043
rs4246215	FEN1	binding	11	C>A	dom	2.51(1.03–6.10)	0.043

* add, additive

dom, dominant

rec, recessive

^#^ Adjusted for age, sex, clinical stage, performance status, smoking status, radiation therapy type, radiation treatment type and dose and lung function

^ p value <0.05 and q value< 0.1.

To evaluate the potential cumulative effects of these SNPs, we selected top SNPs (p<0.01) and performed unfavorable genotype (UFG) analysis. *RPS6KB2*:rs10274 (GA+GG), *SMO*:rs1061280 (AA), *NOTCH1*:rs3124591 (AG+GG), and *GPR30*: rs1133043 (CG+GG) were defined as UFGs, and were included in the analysis (Table 3). Significant dose-response effects were identified: with the increase in the number of UFGs, the risk of developing esophagitis (P-trend = p: 1.03×10^−6^) significantly increased accordingly. Compared with patients with 0–1 UFG, those patients with 2 UFGs had 3.33-fold increased risk of esophagitis (OR: 3.33, 95%CI: 1.19–9.33, p: 0.02). Patients with 3 or 4 UFGs had more than a 13-fold increased risk of developing esophagitis (OR: 13.44, 95% CI: 4.40–41.07, *p*: 5.11×10^−6^).

**Table 3:** Cumulative effect of unfavorable SNPs on esophagitis or pneumonitis.

No. unfavorable SNPs	Events	No event	OR(95%CI)	*p*
Esophagitis*				
0–1	14(36.84%)	24(63.16%)	1(reference)	
2	49(52.13%)	45(47.87%)	3.33 (1.19–9.33)	0.02
3–4	79(69.30%)	35(30.70%)	13.44 (4.40–41.07)	5.11×10^−6^
*P* trend			3.00 (1.93–4.67)	1.03×10^−6^
*Pneumonitis*^				
0	37(29.37%)	89(70.63%)	1(reference)	
1	44(43.56%)	57(56.44%)	1.88 (1.02–3.46)	0.044
2	6(85.71%)	1(14.29%)	55.89 (3.69–845.54)	0.004
*P* trend			2.48 (1.43–4.29)	0.001

*rs10274, rs1061280, rs3124591 and rs1133043 were included in the analysis; rs1061285 was dropped for the linkage with rs1061280

^rs720014 and rs7957 were included in the analysis; rs3757 and rs1633445 were dropped for the linkage with rs720014.

### Associations between individual SNPs and radiation-induced acute pneumonitis

In all, 11 SNPs (6 SNPs in microRNA binding sites and 5 SNPs in microRNA processing genes) were significantly associated with pneumonitis (Table 4). The predictive miRNAs that may potentially target the binding site variants are listed in Table B in S1 File. The most significant SNP, rs720014 (in high LD with rs3757 and rs1633445, *r*^2^>0.8), was located in 3’ UTR of *DGCR8* gene. Patients with GG+GA genotype of *DGCR8*:rs720014 had 3.54- fold increased risk of pneumonitis (OR: 3.54, 95% CI: 1.65–7.61, p <0.05). This SNP remained significant associated with pneumonitis after multiple comparison corrections (q<0.1).

**Table 4:** Association of Pneumonitis with the top 11 MicroRNA SNPs.

SNP	Gene	Site	Chr	Allelic change	Model*	OR (95% CI)^#^	*p*
rs720014	DGCR8	processing	22	A>G	dom	3.54(1.65–7.61)	0.001^
rs3757	DGCR8	processing	22	G>A	dom	3.54(1.65–7.61)	0.001^
rs1633445	DGCR8	processing	22	A>G	dom	3.54(1.65–7.61)	0.001^
rs7957	TNFRSF10D	binding	8	A>G	add	2.44(1.25–4.75)	0.009
rs3087833	GEMIN4	processing	17	G>A	add	2.22(1.21–4.07)	0.01
rs724710	BCL2L11	binding	2	G>A	dom	2.6(1.21–5.57)	0.014
rs573010	RNASEN	processing	5	C>A	dom	2.43(1.14–5.20)	0.022
rs2248718	ATP6V1C1	binding	8	G>A	dom	2.42(1.09–5.35)	0.03
rs4037	ALDH18A1	binding	10	G>A	rec	0.25(0.07–0.91)	0.036
rs7669660	ADH5	binding	4	A>G	dom	0.37(0.14–0.96)	0.041
rs16950113	SMAD7	binding	18	A>G	dom	2.96(1.01–8.65)	0.047

* add, additive

dom, dominant

rec, recessive

^#^ Adjusted for age, sex, clinical stage, performance status, smoking status, radiation therapy type, radiation treatment type and dose and lung function

^ q value< 0.1.

Using the same approach as in the esophagitis analysis, we also performed UFG analysis for the top SNPs (p<0.01) identified for pneumonitis. *DGCR8*:rs720014 (GG) and *TNFRSF10D*:rs7957 (GG) were defined as UFGs and were included in the UFG analysis of pneumonitis (Table 3). A significant dose-response effect was also observed for pneumonitis (P trend = 0.001) with increasing number of UFGs. Compared with patients with no UFG, patients with one UFG had a 1.88-fold increased risk of pneumonitis (OR: 1.88, 95% CI: 1.02–3.64, p: 0.044) and patients with two UFGs had more than a 50-fold increased risk of pneumonitis (OR: 55.89, 95% CI: 3.69–845.54, p = 0.004).

### eQTL analysis

**Fig 1:**
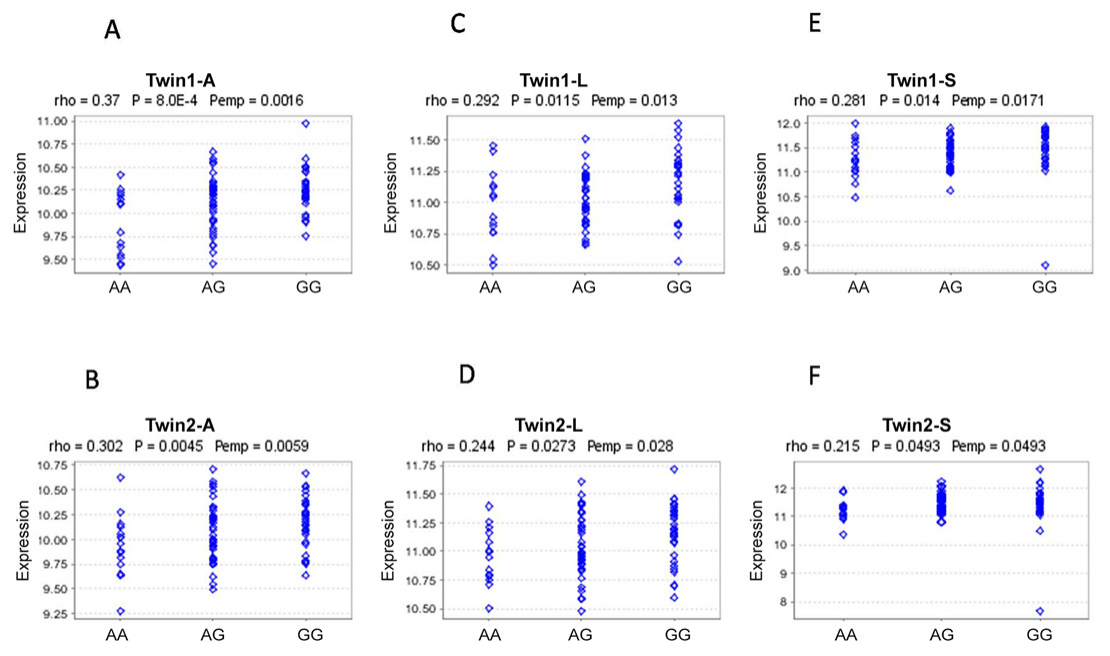
The eQTL analysis of rs10274 based on Genevar Twin study samples. (A) RPS6KB2 expression in subcutaneous fat in Twin1 group; (B) RPS6KB2 expression in subcutaneous fat in Twin2 group; (C) RPS6KB2 expression in lymphoblastoid cell lines in Twin1 group; (D) RPS6KB2 expression in lymphoblastoid cell lines in Twin2 group; (E) RPS6KB2 expression in skin tissues in Twin1 group; (F) RPS6KB2 expression in skin tissues in Twin2 group.

To explore the potential function and underlying mechanism for identified loci, we performed expression quantitative trait loci (eQTL) analysis using an online bioinformatics tool. All 27 SNPs significant for esophagitis (16 SNPs) and pneumonitis (11 SNPs) were entered into the analysis. Genevar showed that the genotype of rs10274 was associated with altered expression of the *RPS6KB2* gene based on data from the Multiple Tissue Human Expression Resource (MuTHER) Study, which analyzed gene expression in adipose and skin tissues as well as lymphoblastoid cell lines (LCLs) derived from 196 Caucasian female twins split into two groups. Compared with the GG genotype, the AA genotype of rs10274 was associated with lower expression of the *RPS6KB2* gene in all tissue types (adipose, skin, and LCLs); the correlation coefficient (rho) ranged from 0.215 to 0.37, and the P value ranged from 0.049 to 8.0×10^−4^ (Fig 1A–1F).

### Gene-based analysis

Because multiple SNPs were analyzed for each gene within the miRNA processing pathway, to summarize the total effects contributed by SNPs within a single gene, we performed a gene-based analysis using VEGAS [23], which tests the association for multiple SNPs in a pre-defined set and takes into account the linkage disequilibrium structure. This software performed simulations using a less computationally intensive Monte Carlo approach. We observed that *DGCR8* (p value: 0.010) and *GEMIN4* (p value: 0.039) were significantly associated with pneumonitis, while *XPO5* reached borderline significance (P = 0.087). However, only *DGCR8* remained significant after adjustment for multiple testing at false discovery rate (FDR) of 10%). *GEMIN4* was the only gene that reached borderline significance for esophagitis (P = 0.065) (Table 5).

**Table 5:** Relationship between the microRNA processing gene and radiation toxicity.

			Esophagitis	Pneumonitis
Chr	Gene	nSNPs	*P* value	*P* value
22	DGCR8	13	0.629	0.01*
17	GEMIN4	7	0.065	0.039
6	XPO5	4	0.364	0.087
1	DDX20	5	0.348	0.151
5	RNASEN	27	0.339	0.168
12	RAN	5	0.387	0.503
14	DICER1	11	0.284	0.56

Gene-based test by VEGAS

*significant at FDR of 10%.

## Discussion

We identified genetic variations in miRNA-related genes that were significantly associated with the risk of developing radiation-induced pneumonitis and esophagitis. After multiple comparisons, *RPS6KB2* and *SMO* binding site SNPs remained significantly associated with radiotherapy-induced acute esophagitis, while *TNFRSF10D*:rs7957 was significantly associated with radiotherapy-induced acute pneumonitis. Gene based analysis supported a significant role of *DGCR8* and *GEMIN4* in the risk of pneumonitis. eQTL analysis showed that the top SNP (*RPS6KB2*:rs10274) for esophagitis analysis could potentially affect *RPS6KB2* expression. These results suggest that genetic variants in miRNA-related genes may serve as potential predictive markers of the risk of developing radiation-induced acute toxicities.

In our analysis, we found that *RPS6KB2*:rs10274 was the most significant SNP associated with the risk of esophagitis. The *RPS6KB2* gene encodes for protein S6 kinase 2 (S6K2), which affects many cellular processes such as cell proliferation, survival, and metastasis. *RPS6KB2* was also reported to have a role in acute radiation effects via the AKT/mTOR pathway [30–32]. *RPS6KB2* was found to be the direct target of mir*-193a-3p* [31]. We found that the AA genotype of *RPS6KB2*:rs10274 had a protective effect against the risk of esophagitis, and cis-eQTL analysis further supported that the AA genotype of rs10274 is associated with lower expression of the *RPS6KB2* gene, while the GG genotype of rs10274 was associated with higher expression of *RPS6KB2*. Although not yet reported in the literature, it is likely that the A genotype of *RPS6KB2*:rs10274 itself or SNP tagged by it could create a new miRNA binding site for miR-193a regulation, which would enable the down regulation of miR-193a-3p on the host gene. The decreased level of *RPS6KB2* expression in turn would protect the cell from radiation-induced toxicity through the AKT/mTOR pathway. Two SNPs (rs1061280 and rs1061285) in the *SMO* gene were also identified as significantly associated with the risk of esophagitis after multiple comparison corrections. Smoothened *(SMO)* encoded a protein that belongs to the G-protein–coupled receptor superfamily. As an important member of the hedgehog (Hh) pathway, this protein SMO was reported to have a driver role in the carcinogenesis of esophagus [33]. It is likely that the binding site SNPs (*SMO*: rs1061280, *SMO*: rs1061285) could affect *SMO* gene transcription or functions that contribute to the etiology of esophagitis after radiotherapy.

*TNFRSF10D*: rs7957 is the most significant binding site SNP identified for pneumonitis. *TNFRSF10D* belongs to the tumor necrosis factor receptor superfamily. *TNFRSF10D* can trigger activation of the AKT pathway [34], which contributes to acute radiation response and acute radiation toxicity [28]. We found that the GG genotype of rs7957 is associated with a higher risk of radiotherapy-induced pneumonitis and at the same time it confers a longer survival time (data not shown). This result may suggest that patients with a high risk of radiation toxicity are those who respond well to radiation. Biologically, the association between *TNFRSF10D* and radiation outcomes may be attributed to the activation of stem cell signaling via TNFRSF10D-activated AKT pathway. Activating stem cell signal pathways will start the repair of radiotherapy-induced cell necrosis or apoptosis [35,36]; at the same time this activation will also promote the proliferation of cancer stem cells, which is the cause of cell resistance to therapy and of metastasis [37]. Consistent with this theory, the GG genotype of *TNFRSF10D*: rs7957 indicates higher risk of pneumonitis and longer survival duration, probably due to the activation of the stem cell pathway- AKT pathway. The role of stem cell signaling pathways in radiotherapy-induced side effects have not been reported. Our data suggest that the AKT/mTOR and SMO-Hh pathway probably contribute to radiotherapy-induced esophagitis, while the AKT pathway probably contributes to the pneumonitis.

By summarizing the effects from multiple SNPs in the same gene, the *DGCR8* and *GEMIN4* are the top genes identified for pneumonitis at the single gene level. *DGCR8* is an important gene that participates in microRNA processing [38] and helps in generating RNA hairpins known as pre-microRNA. Daniel Gomez-Cabello [39] found that *DGCR8* may participate in pneumonitis through affecting fibroblasts ‘cell proliferation. Our results show that three SNPs in the *DGCR8* gene are among the top SNPs identified in pneumonitis, and gene-based analysis showed that the *DGCR8* gene is the most significant gene in the analysis of pneumonitis (P = 0.010). *GEMIN4* is another important microRNA processing gene that interacts with microRNA and forms a ribonucleoprotein to form the RNA-induced silencing complex [40]. *GEMIN4* contributes to carcinogenesis in many cancers, such as kidney [41] and ovarian cancer [42]. Here, we report that the AA genotype of *GEMIN4*: rs3087833 would confer a higher risk of radiation-induced pneumonitis.

This study is the first to comprehensively evaluate the effect of miRNA-related genetic variants on the risk of developing radiation-induced toxicities. However, our study also had a few limitations. First, because of the limited sample size and the paucity of comparable studies available, we did not include a validation step; instead, we performed multiple comparison corrections, which reduced the likelihood of false discovery. Nerveless, independent studies will be warranted to confirm our findings. Second, although eQTL analysis suggests that one of the SNPs may affect gene expression, the biological mechanisms underlying the association of these SNPs with radiation outcome are unclear. Mechanistic studies are needed to clarify the functional impact of the SNPs.

In conclusion, we have provided strong evidence that microRNA-related SNPs can contribute to the prediction of radiation-induced esophagitis and pneumonitis. eQTL analysis further suggests that microRNA binding site SNPs could affect miRNA regulation of host gene expression and thus influence the risk of developing radiotherapy-induced toxicities. Since radiotherapy is important for lung cancer patients, especially those with locally advanced NSCLC, our findings may assist in the customized planning of radiation dose based on the patients’ risk of developing toxicities prior to treatment, thus maximizing treatment effects while minimizing toxicities that are sometimes life threatening.

## Supporting Information

S1 File Potential targeting miRNAs for the significant miRNA binding site SNPs associated with esophagitis (Table 2) (Table A). Potential targeting miRNAs for the significant miRNA binding site SNPs associated with pneumonitis (Table 4) (Table B).
(DOCX)

S1 Table Logistic regression analysis of 233 miRNA-related SNPs and risk of esophagitis in locally-advanced lung cancer patients. (XLSX)

S2 Table Logistic regression analysis of 233 miRNA-related SNPs and risk of pneumonitis in locally-advanced lung cancer patients. (XLSX)
