# Supplementary material for: MiRNA-Related Genetic Variations Associated with Radiotherapy-Induced Toxicities in Patients with Locally Advanced Non–Small Cell Lung Cancer
Source: PLoS One. 2016 Mar 18;11(3):e0150467. doi: 10.1371/journal.pone.0150467 (PMC4798772; doi:10.1371/journal.pone.0150467)
Supplement: S1 File — Potential targeting miRNAs for the significant miRNA binding site SNPs associated with pneumonitis (Table 4) (Table B). (DOCX) [file pone.0150467.s001.docx]

| **Table A. Potential targeting miRNAs for the significant miRNA binding site SNPs associated with esophagitis (Table 2)** | | |
| --- | --- | --- |
| **SNP** | **Gene** | **Targeting miRNA*** |
| rs10274 | RPS6KB2 | miR-4455 , miR-644a , miR-6747-5p, miR-1268a , miR-1268b ,  miR-744-5p |
| rs1061280 | SMO | miR-1322, miR-143-3p, miR-4770, miR-6088 |
| rs1061285 | SMO | miR-3120-5p, miR-370-3p, miR-626, miR-6876-3p, miR-6893-3p,  miR-3160-5p, miR-3192-3p, miR-4762-3p |
| rs3124591 | NOTCH1 | miR-2116-5p, miR-22-5p, miR-362-5p, miR-4273  miR-4677-5p, miR-500a-5p, miR-500b-5p, miR-6739-3p, miR-7156-5p |
| rs1133043 | GPR30 | miR-1199-5p , miR-146a-3p , miR-27b-5p , miR-301a-5p  miR-3921 , miR-4303 , miR-4653-5p , miR-6751-3p  miR-6817-3p , miR-6873-3p , miR-7110-3p, miR-615-3p ,  miR-6845-3p |
| rs16950113 | SMAD7 | miR-1206, miR-4729, miR-5696, miR-579-3p, miR-664b-3p, miR-6853-3p, miR-302a-5p |
| rs713065 | FZD4 | miR-494-3p |
| rs2075993 | E2F2 | miR-1207-5p, miR-3918, miR-4514, miR-4692, miR-4763-3p, miR-6132, miR-6808-5p, miR-6836-5p, miR-6893-5p, miR-6895-5p, miR-940, miR-1908-5p, miR-3937, miR-663a, miR-6762-5p, miR-6787-5p, miR-6845-5p  miR-744-5p |
| rs7588 | PLK1 | miR-152-5p, miR-182-3p, miR-218-2-3p, miR-597-3p |
| rs1052133 | OGG1 | miR-1908-5p, miR-663a , miR-6787-5p , miR-1256 , miR-4467 , miR-6770-3p |
| rs4690150 | FGF5 | miR-618, miR-6844, miR-452-5p, miR-4676-3p, miR-8068 , miR-892c-3p |
| rs2248718 | ATP6V1C1 | miR-147a, miR-3911 , miR-644a |
| rs4246215 | FEN1 | miR-6818-3p, miR-3149, miR-3713 |

* Based on in silico prediction by PolymiRTS v3.0

| **Table B. Potential targeting miRNAs for the significant miRNA binding site SNPs associated with pneumonitis (Table 4)** | | |
| --- | --- | --- |
| **SNP** | **Gene** | **Targeting miRNA*** |
| rs7957 | TNFRSF10D | miR-1199-5p , miR-146a-3p , miR-4303 , miR-6751-3p, miR-615-3p |
| rs724710 | BCL2L11 | miR-323b-3p, miR-515-5p, miR-519d-5p, miR-519e-5p, miR-5695 |
| rs2248718 | ATP6V1C1 | miR-147a, miR-3911, miR-644a |
| rs4037 | ALDH18A1 | miR-498, miR-7112, miR-499a-3p, miR-499b-3p, miR-607, miR-6715b-3p |
| rs7669660 | ADH5 | miR-5579-5p, miR-584-5p |
| rs16950113 | SMAD7 | miR-1206, miR-4729, miR-5696, miR-579-3p, miR-664b-3p, miR-6853-3p, miR-302a-5p |

* Based on in silico prediction by PolymiRTS v3.0
